# Supplementary material for: Circular single-stranded DNA as a programmable vector for gene regulation in cell-free protein expression systems
Source: Nat Commun. 2024 May 31;15:4635. doi: 10.1038/s41467-024-49021-6 (PMC11143192; doi:10.1038/s41467-024-49021-6)
Supplement: Supplementary file 1 — Supplementary Information [file 41467_2024_49021_MOESM1_ESM.pdf]

Supplementary Information for

Circular single-stranded DNA as a programmable vector for gene regulation in cell-free protein expression systems

Zhijin Tian[1,2]†, Dandan Shao[3]†, Linlin Tang[2,3]†, Zhen Li[3], Qian Chen[4], Yongxiu Song[2,5], Tao Li[1], Friedrich C. Simmel[6], Jie Song[2,3] *

[1]Department of Chemistry, University of Science & Technology of China, Hefei, Anhui, 230026, China.

[2]Hangzhou Institute of Medicine, Chinese Academy of Sciences, Hangzhou, Zhejiang 310022, China.

[3]Institute of Nano Biomedicine and Engineering, Department of Instrument Science and Engineering, School of Electronic Information and Electrical Engineering, Shanghai Jiao Tong University, Shanghai 200240, China.

[4]College of Forestry, Northeast Forestry University, Harbin 150040, Heilongjiang, China.

[5]Ningbo institute of Dalian University of Technology, Ningbo 315016, China.

[6]Department of Bioscience, School of Natural Sciences, Technische Universität München, Garching, Germany

*Corresponding authors. Email: songjie@him.cas.cn

†These authors contributed equally to this work.

This PDF file includes:

Supplementary Figures 1-28

Supplementary Tables 1-4

Supplementary Figures

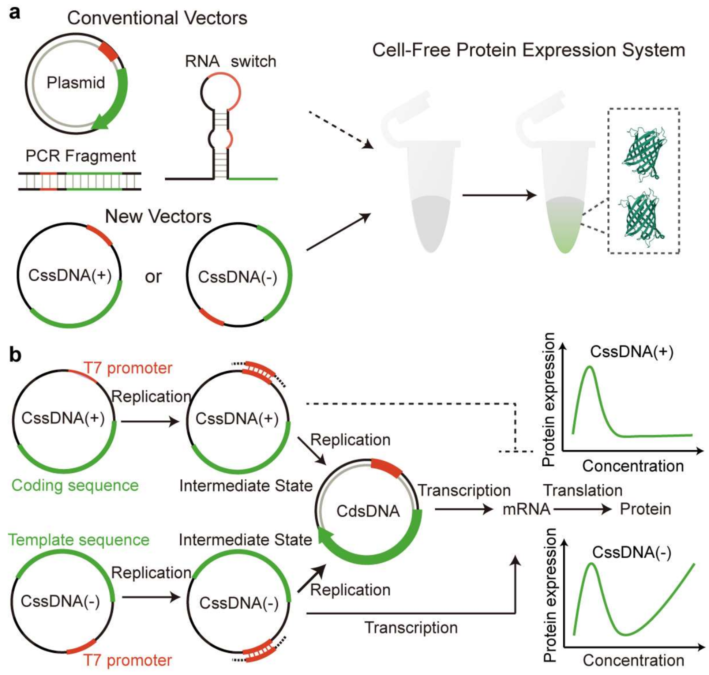

Supplementary Figure 1. Summary of the manuscript. **a** Circular single-stranded DNA (CssDNA) can serve as a programmable vector for gene regulation in cell-free protein expression (CFE) systems; **b** The differing expression fates of sense CssDNA (+) and antisense CssDNA (-) in CFE systems are determined.

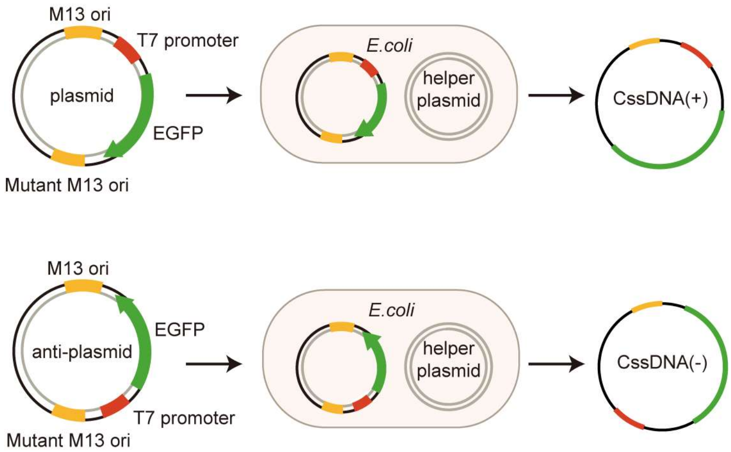

Supplementary Figure 2. Schematic of phagemid production for generating circular single-stranded DNA and the features of sense/antisense plasmids, as well as the corresponding CssDNA.

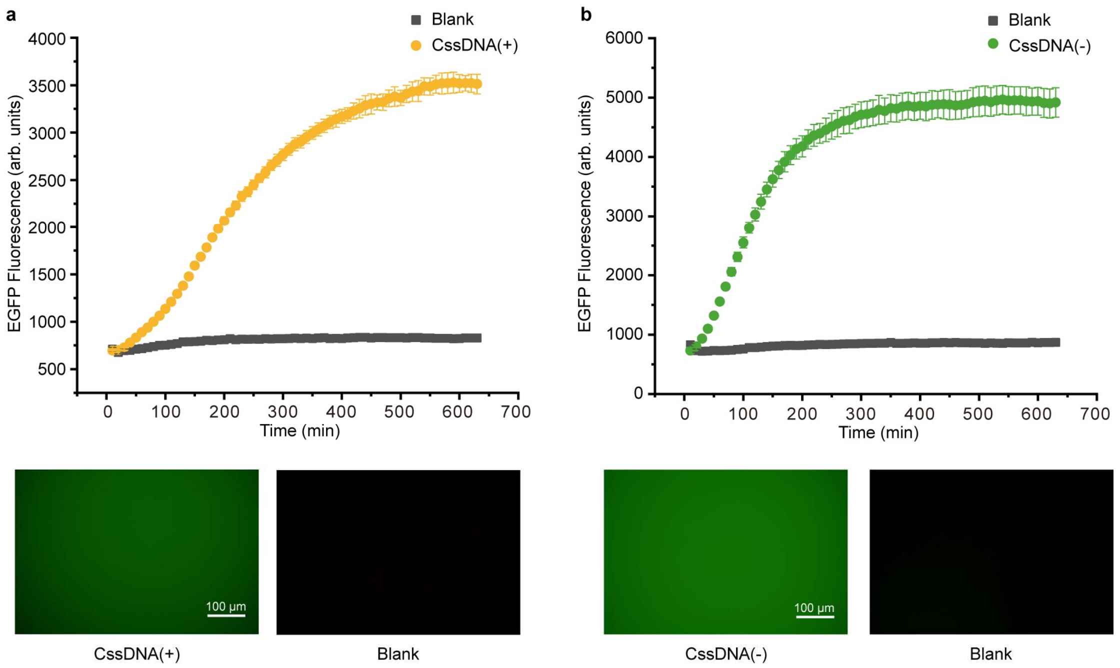

Supplementary Figure 3. Protein expression kinetic curves of CssDNA(+) (**a**) and CssDNA(-) (**b**) before normalization. And the fluorescence images of CssDNA at a concentration of 5 ng/μL, at which point protein expression had reached a plateau. The images were compared to a blank group. Data collected in **a** and **b** were monitored by a microplate reader and are presented as mean ± standard deviation (s.d.) for n = 3 biologically independent experiments, source data provided. All images are representative of one of n = 3 biologically independent experiments; similar results were observed each time. Scale bar 100 μm.

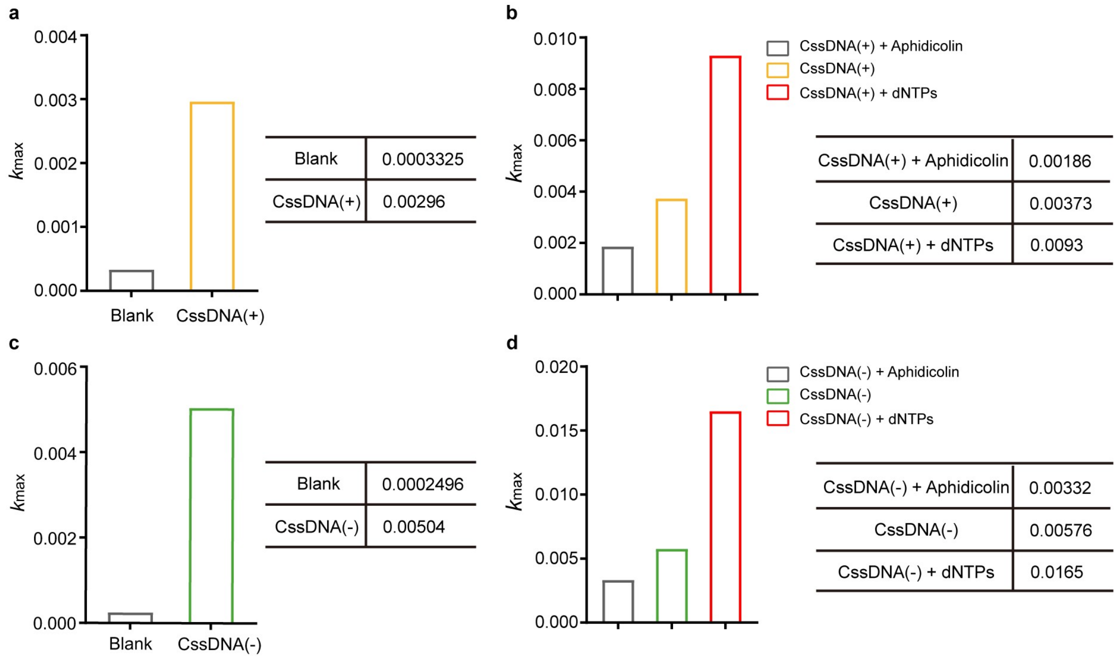

Supplementary Figure 4. The maximum rate constants (k_{max}) were obtained by taking the first derivative of the fluorescence kinetic curve. **a**, **c**. Maximum rate constants of the fluorescence kinetic curve of CssDNA(+) (**a**) and CssDNA(-) (**c**). **b**, **d**. Maximum rate constants of the fluorescence kinetic curve of CssDNA(+) (**b**) and CssDNA(-) (**d**) in the presence of aphidicolin or

dNTPs.

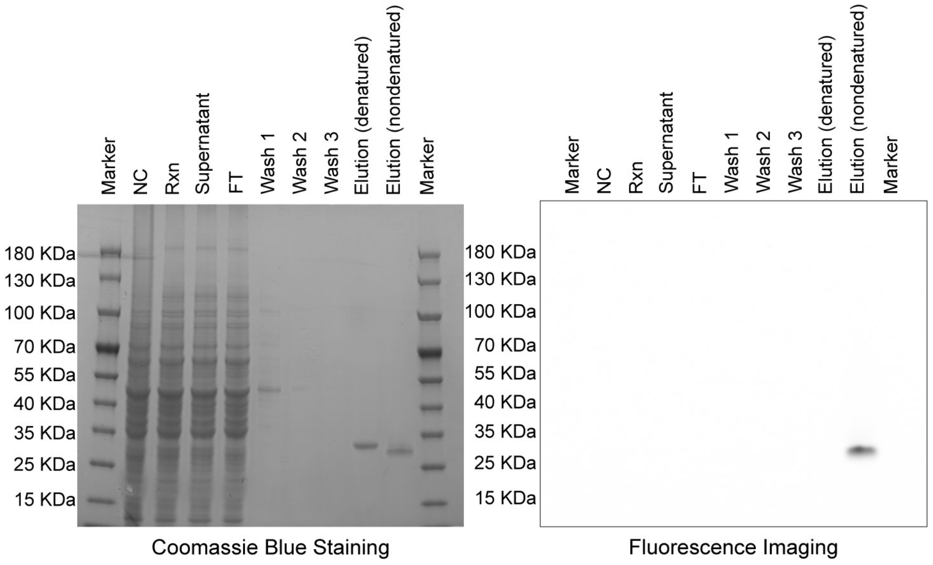

Supplementary Figure 5. Characterization of protein purification process. The gels of coomassie blue staining and fluorescence imaging showed that the expression EGFP was purified. NC: blank reaction mixture as negative control. Rxn: reaction mixture containing proteins expressed by CssDNA template. Supernatant: supernatant obtained by Rxn centrifugation. FT: liquid flowing through magnetic beads. Wash 1-3: liquid after washing magnetic beads 1-3 times. Elution (denatured): the proteins in elution buffer was denatured by heating. Elution (nondenatured): the proteins in elution buffer wasn't denatured.

The purified protein was quantified by bicinchoninic acid (BCA) assay after overnight dialysis. The result showed that when the final concentration of CssDNA(+) was 1 ng/μL, the protein yield was 10 mg/L.

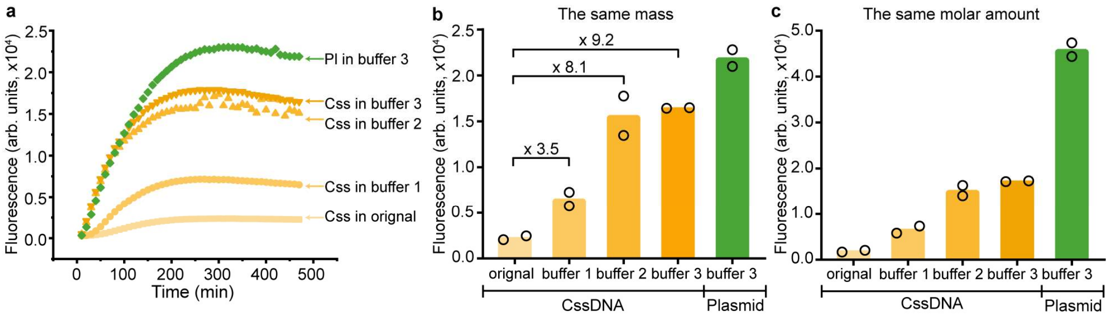

d

Components / System	Commercial system	Tirs-HCl (4.17 mM)	$MgCl_2$ (0.83 mM)	DTT (0.33 mM)	$(NH_4)_2SO_4$ (0.83 mM)	BSA (0.17 mM)	Primer (8.3 nM)	dNTPs	Phi29 DNA polymerase (16.7 units/mL)
Original	√	–	–	–	–	–	–	–	–
Buffer 1	√	√	√	√	√	√	√	60 μM	–
Buffer 2	√	√	√	√	√	√	√	60 μM	√
Buffer 3	√	√	√	√	√	√	√	175 μM	√

Supplementary Figure 6. Comparison of expression level of CssDNA and plasmid template and optimization of the reaction system. **a.** Changes in protein expression levels over time for CssDNA and its corresponding plasmid under different reaction systems. Data collected in **a** were monitored

using a microplate reader and are presented as mean values. All templates have the same mass (50 ng). **b, c.** Comparison of fluorescence intensity of EGFP produced by CssDNA and plasmid of the same mass (50 ng) and the same molar amount (0.1 pmol) in different reaction systems. Data collected in **b** and **c** are presented as means with individual data points overlaid, representing $n = 2$ biologically independent experiments. Source data are provided as a Source Data file. **d.** The components of different reaction systems.

We optimize three reaction systems based on the original commercial system by adding additional components to speed up the DNA replication, namely buffer 1, buffer 2 and buffer 3. We compare the expression levels of CssDNA and plasmid of the same mass and the same molar amount in different reaction systems, respectively, as shown in Supplementary Fig. 6a-c. Although the expression level of CssDNA has not yet reached that of the plasmid, after our optimization, the protein expression level of CssDNA in the final reaction system (buffer 3) has been increased almost 10 times compared to the original commercial buffer, narrowing the gap with the plasmid to some extent. The additional components added to the different reaction systems are tabulated in Supplementary Fig. 6d.

The expression level of CssDNA in the current optimized cell free systems is still not better than the state of art with dsDNA, but we believe it might be further improved in the future. As a closed reaction system, the in vitro expression system only has a finite amount of substrate, its components degrade over time, and it does not allow for exchange of material and energy, which limits overall protein expression. Conversely, inside living cells, there is a continuous supply of matter and energy. Our previous work has compared the expression of CssDNA and plasmids in different mammalian cells and found that in many cells, the expression level of CssDNA can be comparable to or even exceed that of plasmids[1]. We therefore believe that extending the lifetime of the cell-free expression reaction, and potentially operating in an open reactor might also bring up expression levels from CssDNA further.

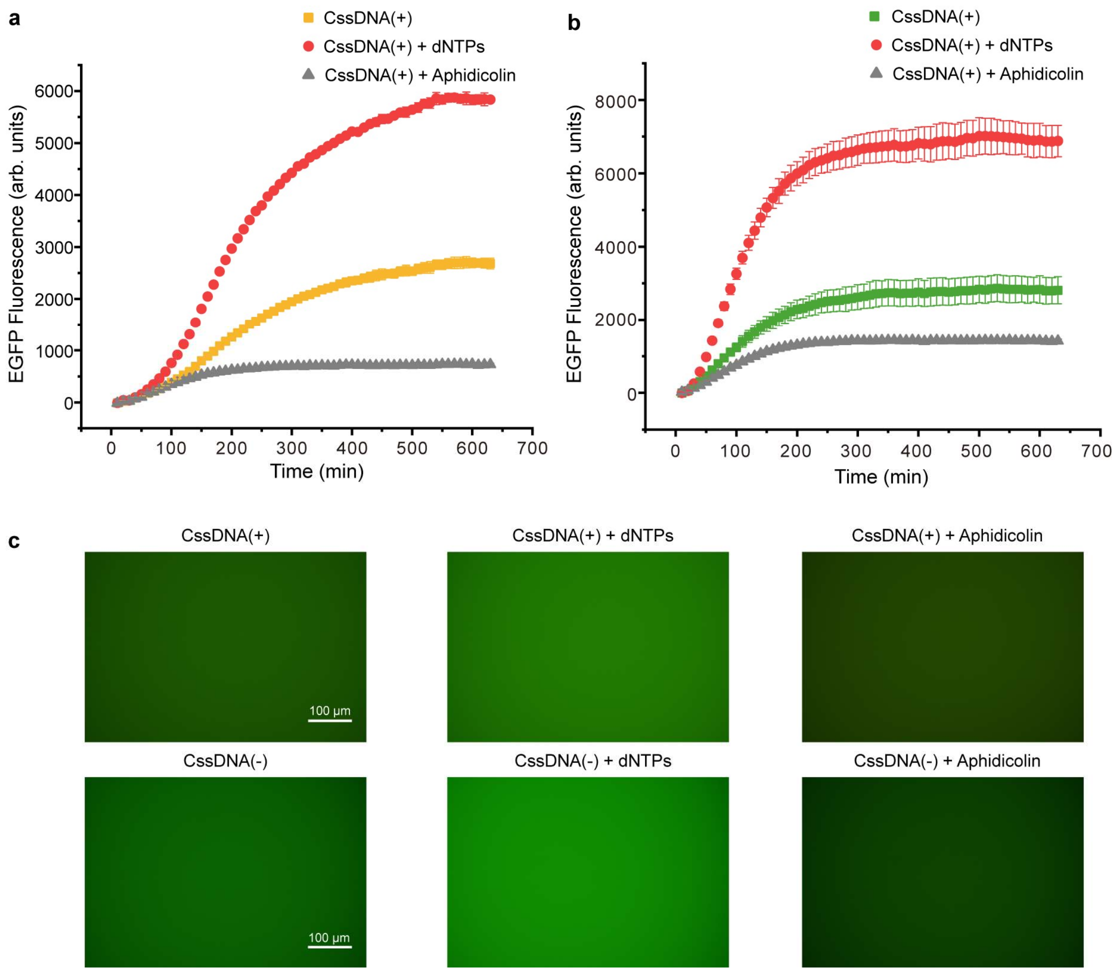

Supplementary Figure 7. Effect of additives, such as dNTPs and aphidicolin, on CssDNA protein expression. **a**, **b**. In the presence of dNTPs or aphidicolin, protein expression kinetic curves of CssDNA(+) (**a**) and CssDNA(-) (**b**) before normalization. And the fluorescence images of CssDNA(+) and CssDNA(-) at a concentration of 5 ng/μL were observed when protein expression had reached a plateau. The images were compared to the control group of CssDNA alone. Data collected in **a** and **b** were monitored by a microplate reader and are presented as mean ± standard deviation (s.d.) for *n* = 3 biologically independent experiments. Source data are provided as a Source Data file. All images are representative of one of *n* = 3 biologically independent experiments; similar results were observed each time. Scalebar 100 μm.

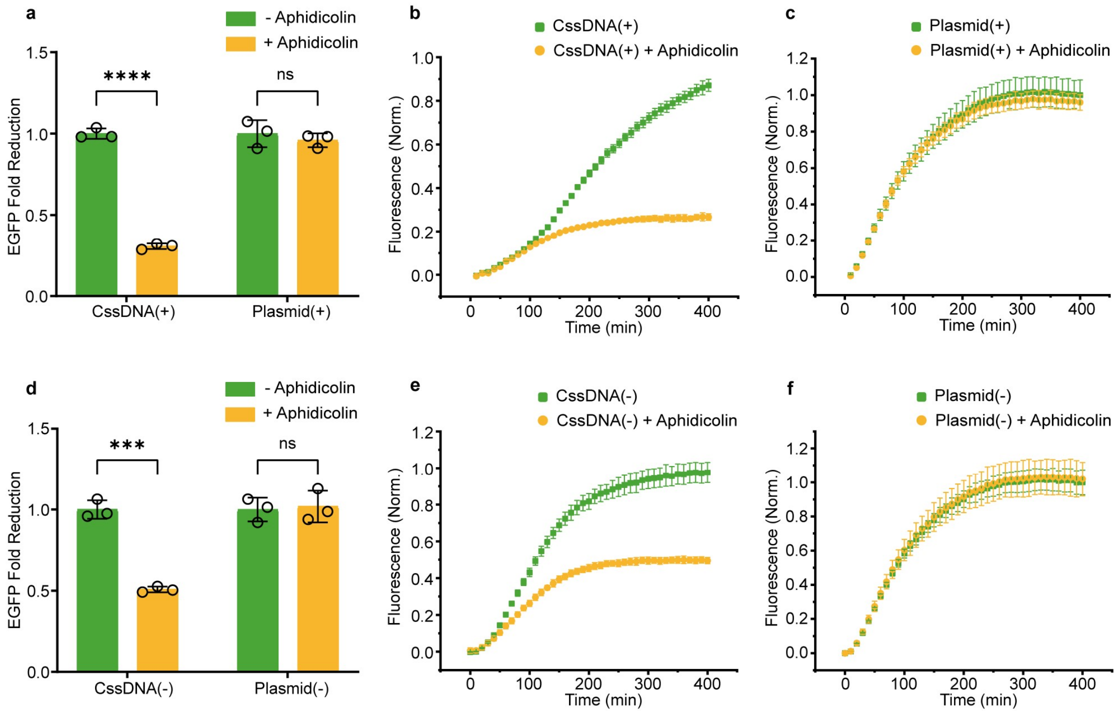

Supplementary Figure 8. Effect of aphidicolin on protein expression of the CssDNA and plasmid vectors. **a**, **d**. Effect of aphidicolin on expression level of CssDNA(+) and plasmid(+) (**a**), CssDNA(-) and plasmid(-) (**d**). **b**, **c**. Changes in protein expression levels over time for CssDNA(+) (**b**) and plasmid(+) (**c**) vectors in the presence of aphidicolin. **e**, **f**. Changes in protein expression levels over time for CssDNA(-) (**e**) and plasmid(-) (**f**) vectors in the presence of aphidicolin. The final concentration of different templates is 1 ng/μL. All fluorescence signals were normalized according to the fluorescence intensity of the highest expression level of the corresponding expression template. Data were monitored by a microplate reader and are presented as mean ± standard deviation (s.d.) for $n = 3$ biologically independent experiments, individual data points in **a** and **d** are overlaid. Source data are provided as a Source Data file. Statistical analysis was performed using two-tailed Student's *t* test (*$p \leq 0.05$, **$p \leq 0.01$, ***$p \leq 0.001$, ****$p \leq 0.0001$, ns $p > 0.05$).

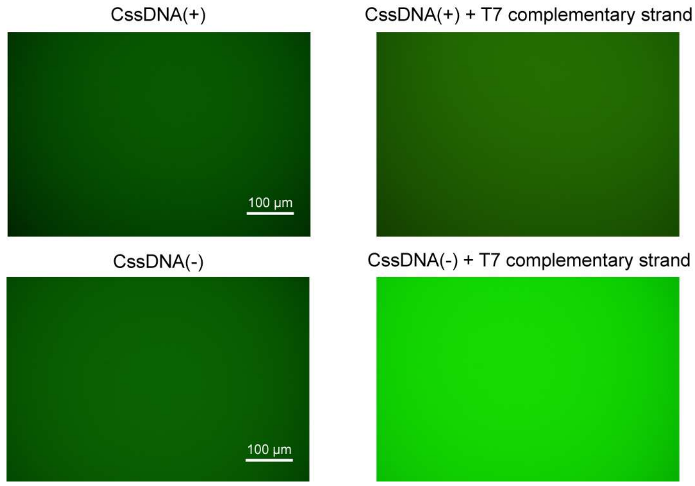

Supplementary Figure 9. Effect of corresponding T7 complementary strand on protein expression of CssDNA. After adding the T7 complementary strand corresponding to CssDNA, fluorescence

images of CssDNA(+) and CssDNA(-) at a concentration of 5 ng/μL were obtained when protein expression had reached a plateau. The images were compared to the control group of CssDNA alone. All images are representative of one of n = 3 biologically independent experiments; similar results were observed each time. Scalebar 100 μm.

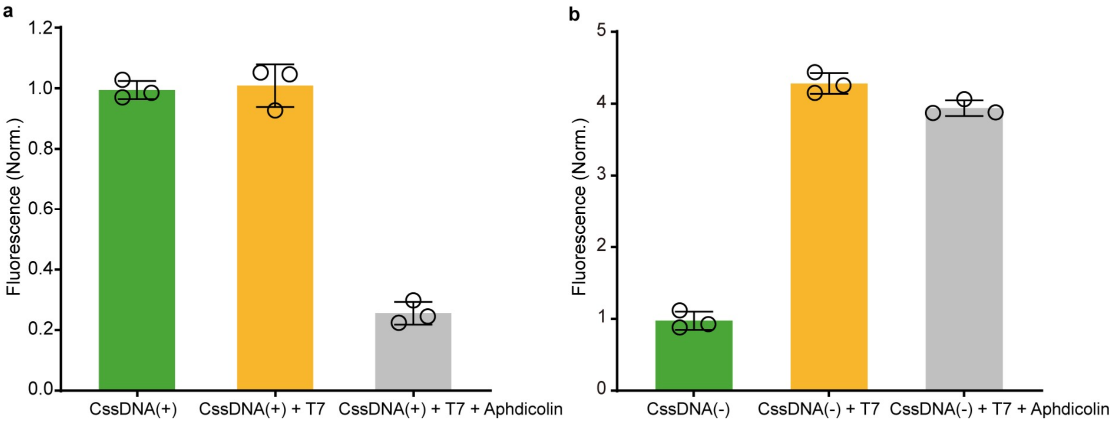

Supplementary Figure 10. Effect of T7 complementary strand and T7 complementary strand coexisting with aphidicolin on CssDNA protein expression. **a.** At a concentration of 5 ng/μL, aphidicolin inhibited CssDNA(+) expression by up to 30% in the presence of T7 complementary strand. **b.** At a concentration of 5 ng/μL, the inhibitory effect of aphidicolin on CssDNA(-) expression was negligible in the presence of the corresponding T7 complementary strand. All fluorescence signals were normalized according to the fluorescence intensity of the corresponding CssDNA expression plateau. Data were monitored by a microplate reader and are presented as mean ± standard deviation (s.d.) for n = 3 biologically independent experiments, individual data points are overlaid. Source data are provided as a Source Data file.

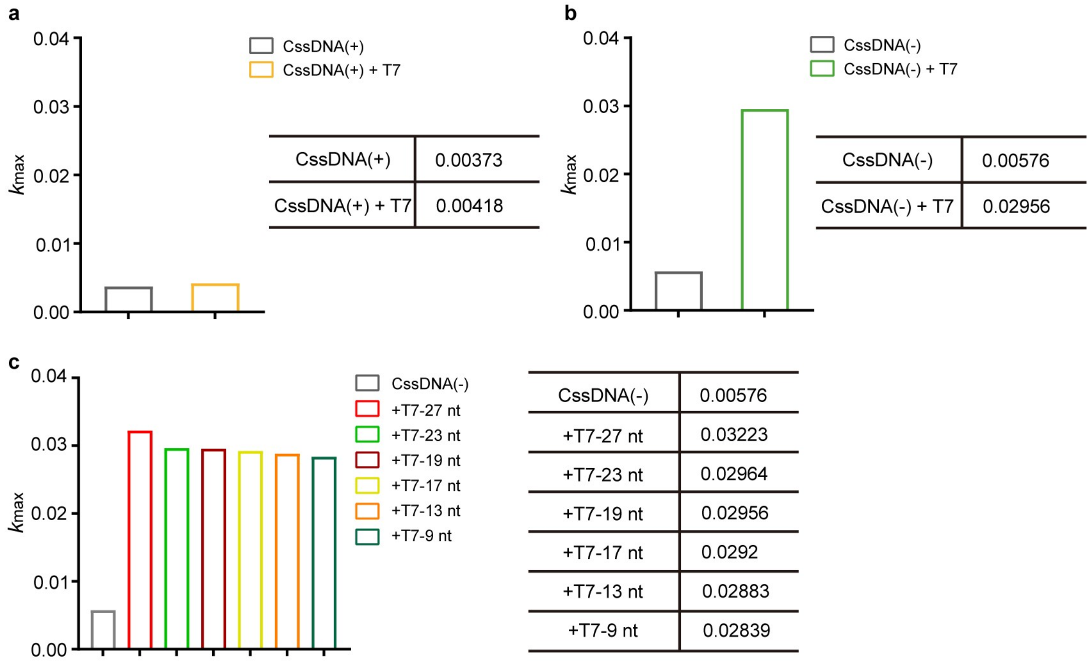

CssDNA(+)	0.00373
CssDNA(+) + T7	0.00418

CssDNA(-)	0.00576
CssDNA(-) + T7	0.02956

CssDNA(-)	0.00576
+T7-27 nt	0.03223
+T7-23 nt	0.02964
+T7-19 nt	0.02956
+T7-17 nt	0.0292
+T7-13 nt	0.02883
+T7-9 nt	0.02839

Supplementary Figure 11. The maximum rate constants (k_{max}) were obtained by taking the first derivative of the fluorescence kinetic curve. **a**, **b**. Maximum rate constants of the fluorescence

kinetic curve of CssDNA(+) (**a**) and CssDNA(-) (**b**) in the presence of T7 complementary strand. **c**. Maximum rate constants of the fluorescence kinetic curve of CssDNA(-) bound to different T7 complementary strands.

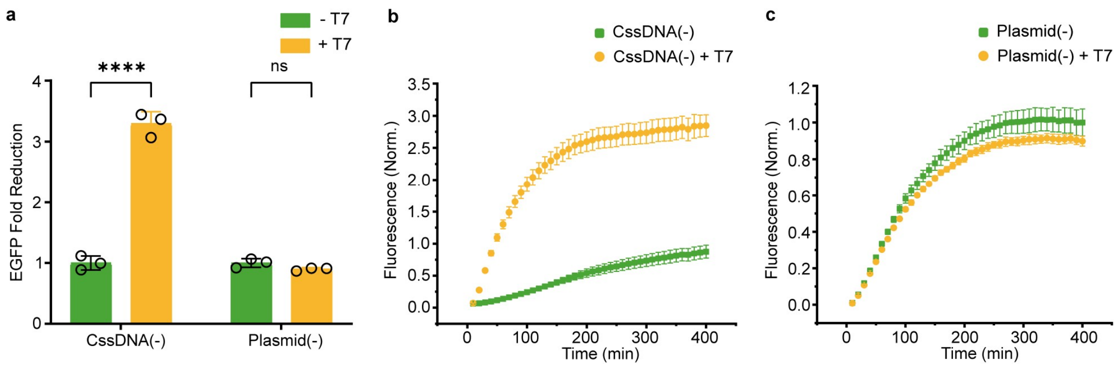

Supplementary Figure 12. Effect of T7 complementary strand on protein expression of the CssDNA(-) and plasmid(-). **a**. Comparison of expression level of CssDNA(-) and plasmid(-) in the presence of T7 complementary strand. **b**, **c**. Changes in protein expression levels over time for CssDNA(-) (5 ng/μL) (**b**) and plasmid(-) (1 ng/μL) (**c**) vectors in the presence of T7 complementary strand. All fluorescence signals were normalized according to the fluorescence intensity of the highest expression level of the corresponding expression template. Data were monitored by a microplate reader and are presented as mean ± standard deviation (s.d.) for n = 3 biologically independent experiments, individual data points in **b** and **c** are overlaid. Source data are provided as a Source Data file. Statistical analysis was performed using two-tailed Student's t test (*p ≤ 0.05, **p ≤ 0.01, ***p ≤ 0.001, ****p ≤ 0.0001, ns p > 0.05).

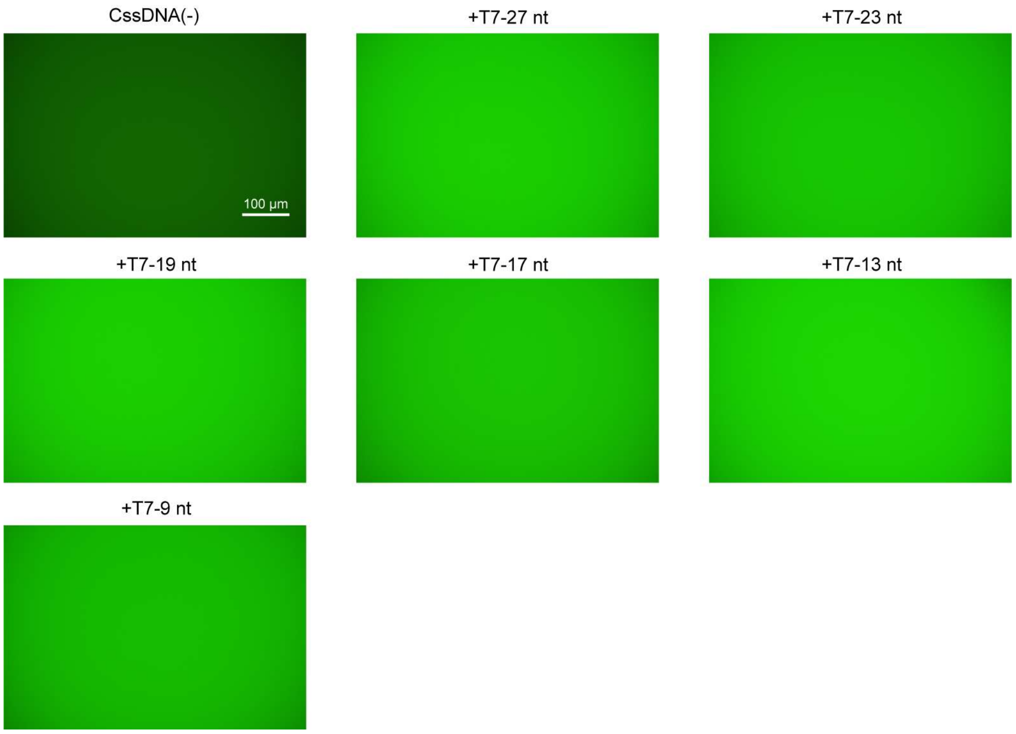

Supplementary Figure 13. Fluorescence images of CssDNA(-) at a concentration of 5 ng/μL after the addition of T7 complementary strands of different lengths at the plateau of protein expression.

All images are representative of one of $n = 3$ biologically independent experiments; similar results were observed each time. Scalebar 100 µm.

a

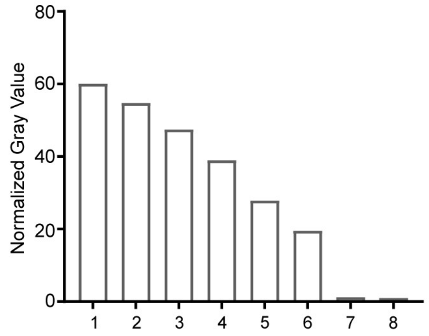

b

	RNA concentration
+T7-27 nt	744.0 ng/µL
+T7-23 nt	457.5 ng/µL
+T7-19 nt	320.5 ng/µL
+T7-17 nt	218.6 ng/µL
+T7-13 nt	184.2 ng/µL
+T7-9 nt	119.9 ng/µL
CssDNA(+)	34.0 ng/µL
CssDNA(-)	21.7 ng/µL

Supplementary Figure 14. Quantification of mRNA transcribed by CssDNA(-) in vitro. **a**. Gray values of the gel electrophoresis bands in Figure 2f were quantified using ImageJ. **b**. The mRNA concentrations measured by nanodrop.

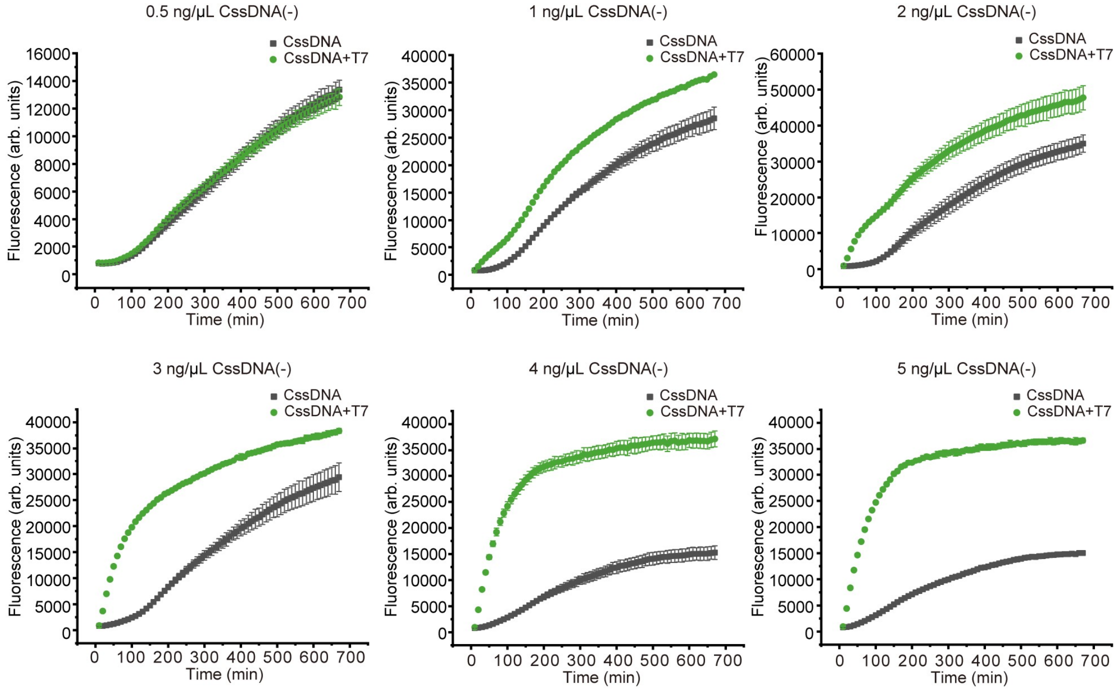

Supplementary Figure 15. Fluorescence signal of CssDNA(-) changes over time as the concentration and the binding to 19-nt T7 complementary strands vary. Data were monitored by a microplate reader and are presented as mean ± standard deviation (s.d.) for $n = 3$ biologically independent experiments. Source data are provided as a Source Data file.

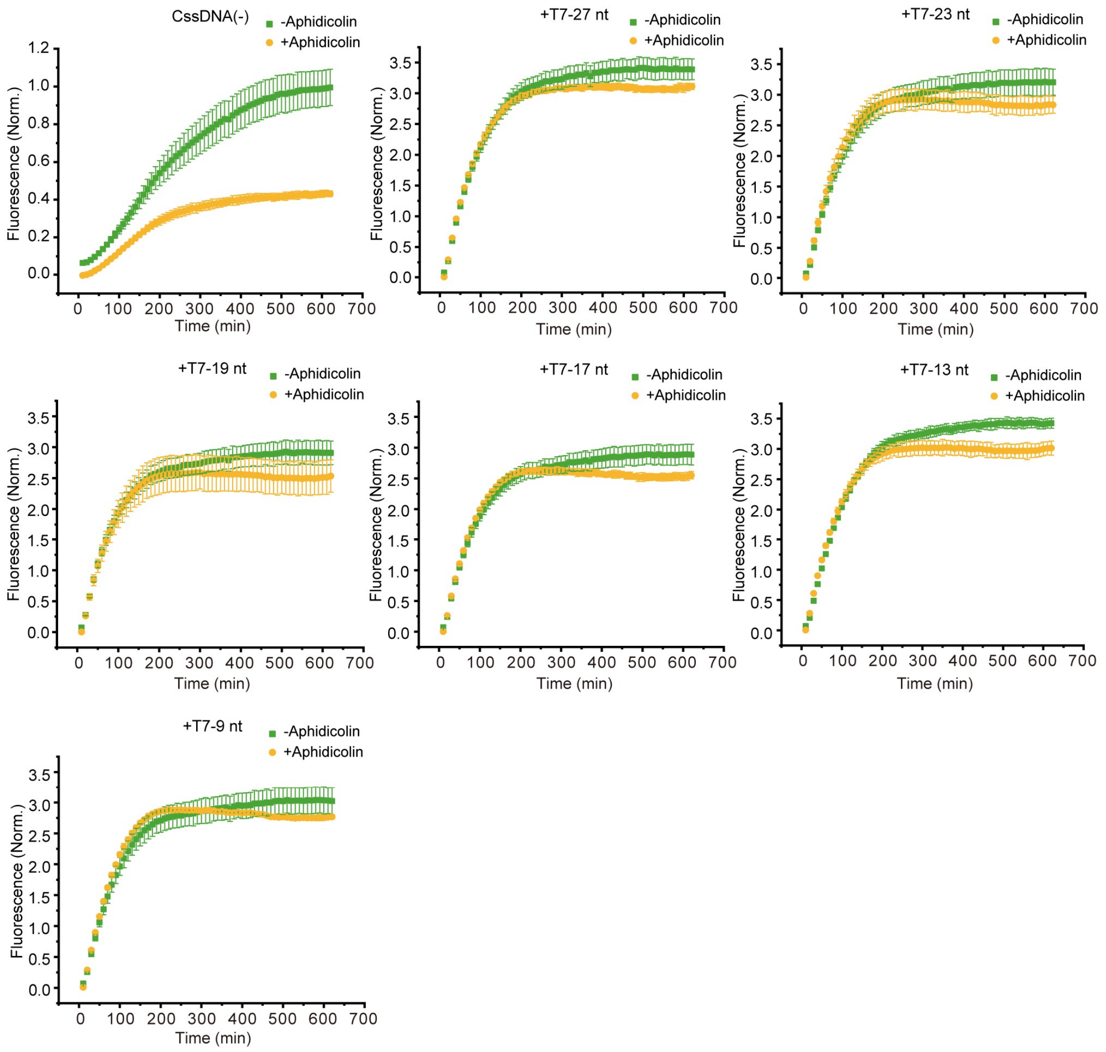

Supplementary Figure 16. The fluorescence signal of 5 ng/μL CssDNA(-) changes over time with and without aphidicolin, both when acting alone and when binding to T7 complementary strands of varying lengths. All fluorescence signals were normalized according to the fluorescence intensity of the highest expression level of the CssDNA(-) vector without aphidicolin. Data were monitored by a microplate reader and are presented as mean ± standard deviation (s.d.) for *n* = 3 biologically independent experiments. Source data are provided as a Source Data file.

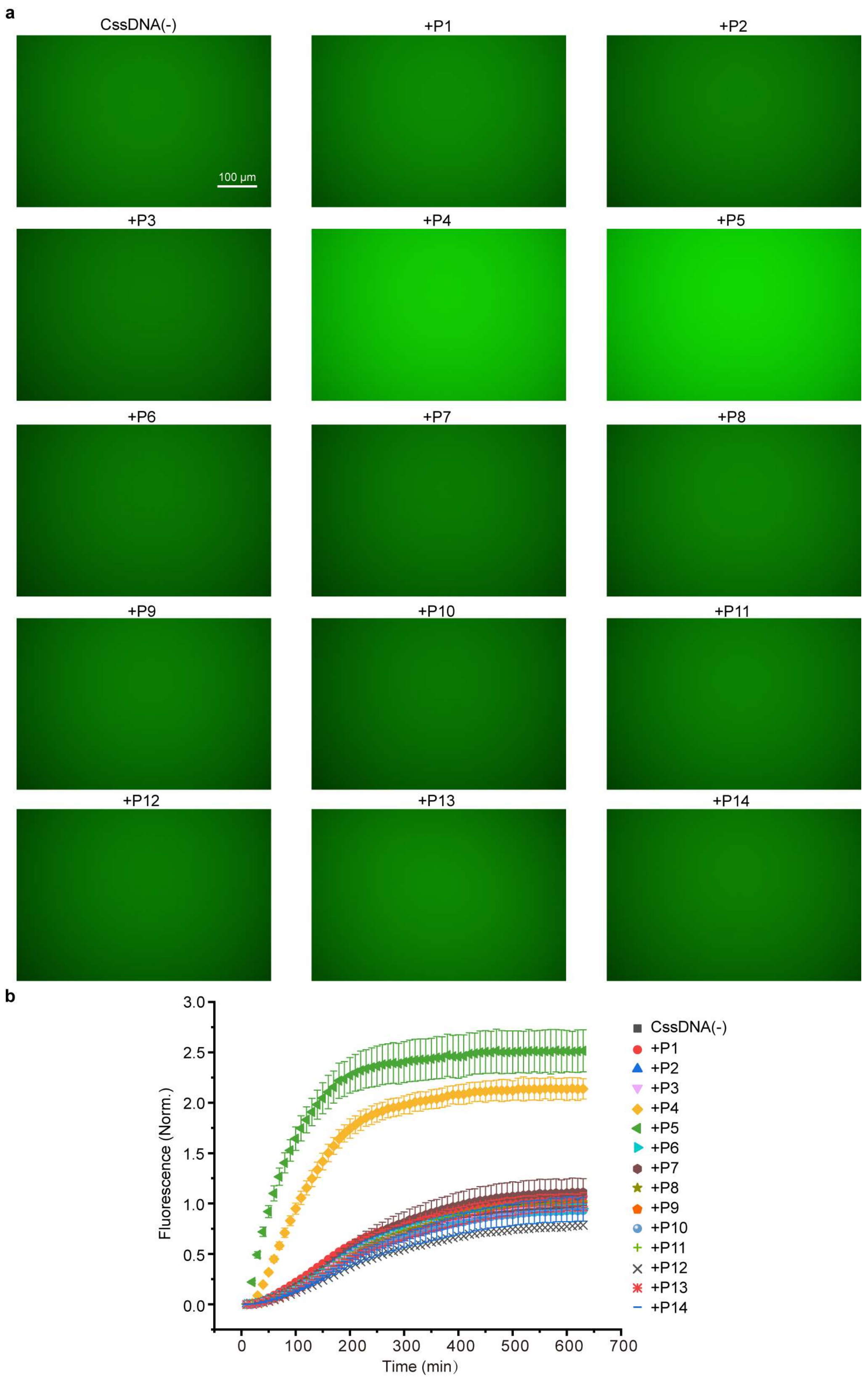

Supplementary Figure 17. Effect of DNA strands complementary to different regions of CssDNA(-) on protein expression. **a.** Fluorescence images of 5 ng/μL CssDNA(-)after the addition of complementary DNA strands, when protein expression reached a plateau. All images are representative of one of n = 3 biologically independent experiments; similar results were observed

each time. Scalebar 100 µm. **b.** Fluorescence signal of CssDNA(-) alone and CssDNA(-) bound to complementary DNA strands in different regions changes over time. All fluorescence signals were normalized according to the fluorescence intensity of the highest expression level of the CssDNA(-) vector without complementary strands. Data were monitored by a microplate reader and are presented as mean ± standard deviation (s.d.) for $n = 3$ biologically independent experiments. Source data are provided as a Source Data file.

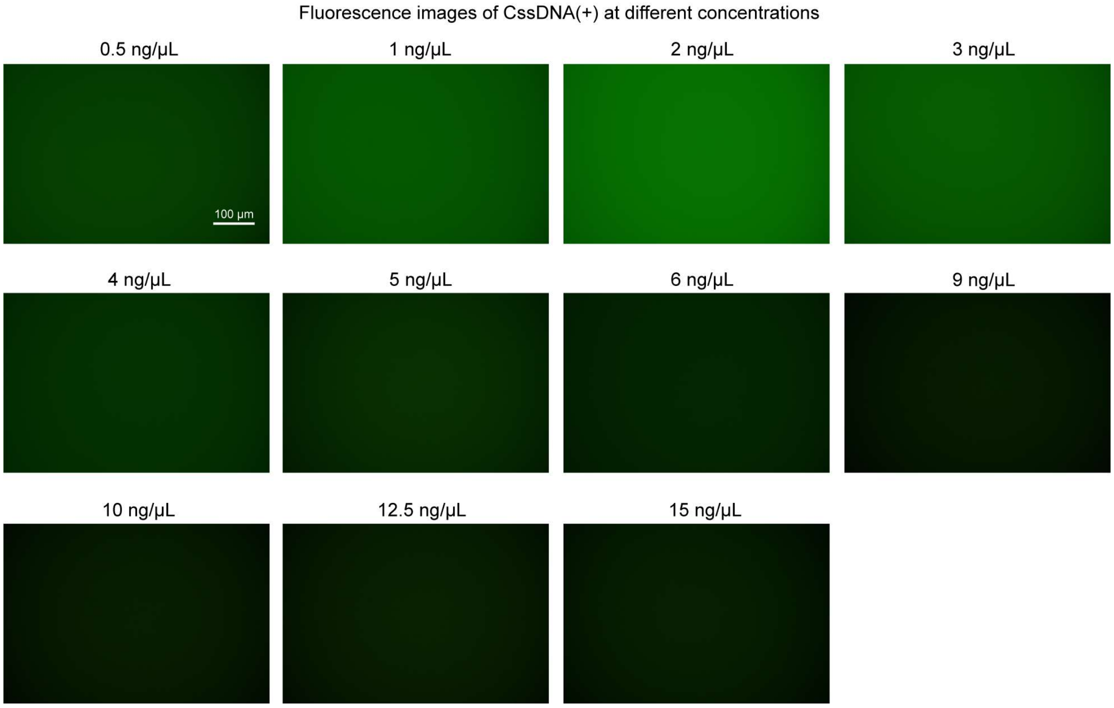

Supplementary Figure 18. Fluorescence images of CssDNA(+) at different concentrations when expression had reached the plateau stage. All images are representative of one of $n = 3$ biologically independent experiments; similar results were observed each time. Scalebar 100 µm.

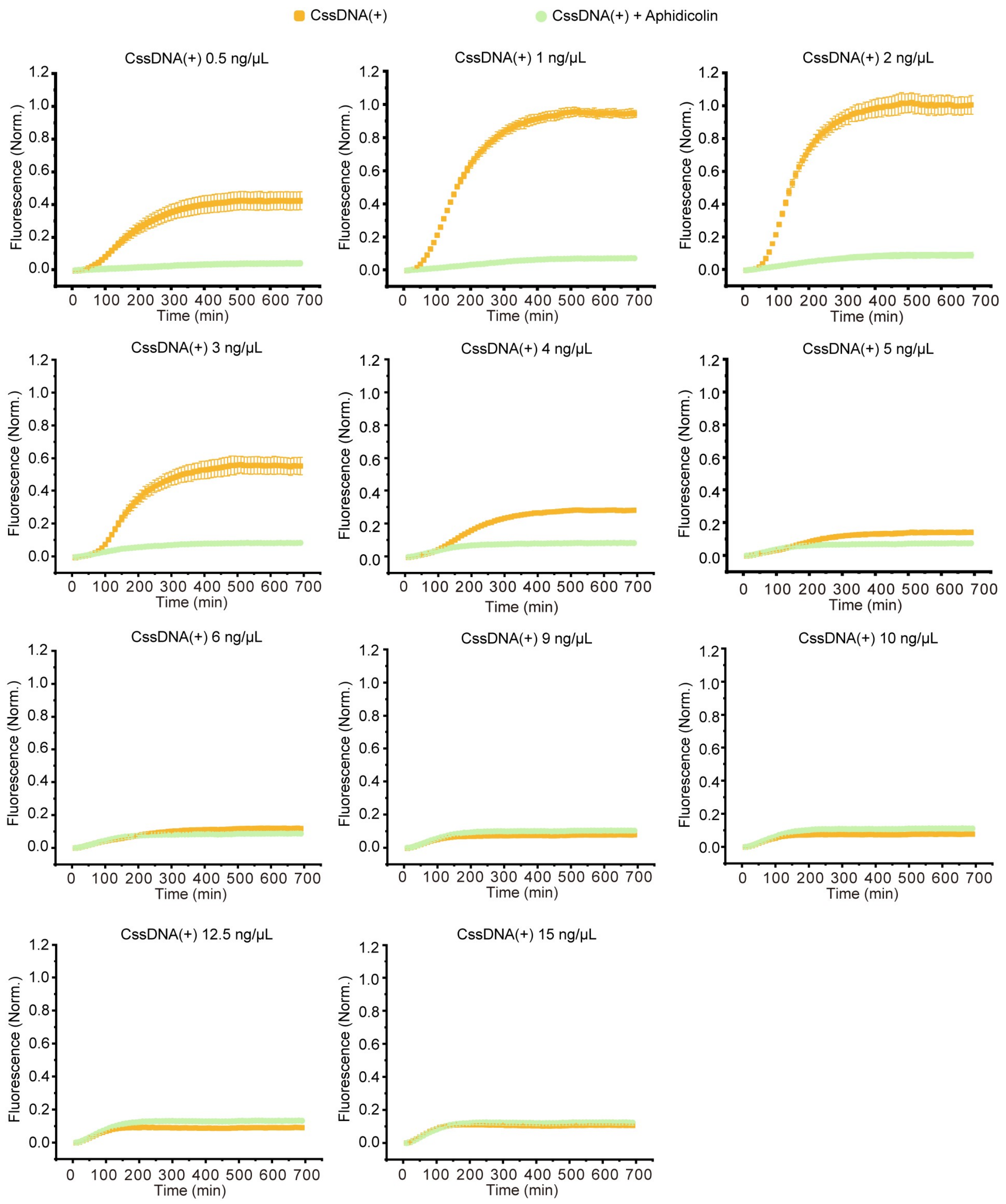

Supplementary Figure 19. Changes of the fluorescence signals of CssDNA(+) at different concentrations over time in the absence and presence of aphidicolin. All fluorescence signals were normalized based on the average fluorescence of CssDNA(+) expression plateau at a concentration of 2 ng/μL. Data were monitored by a microplate reader and are presented as mean ± standard deviation (s.d.) for $n = 3$ biologically independent experiments. Source data are provided as a Source Data file.

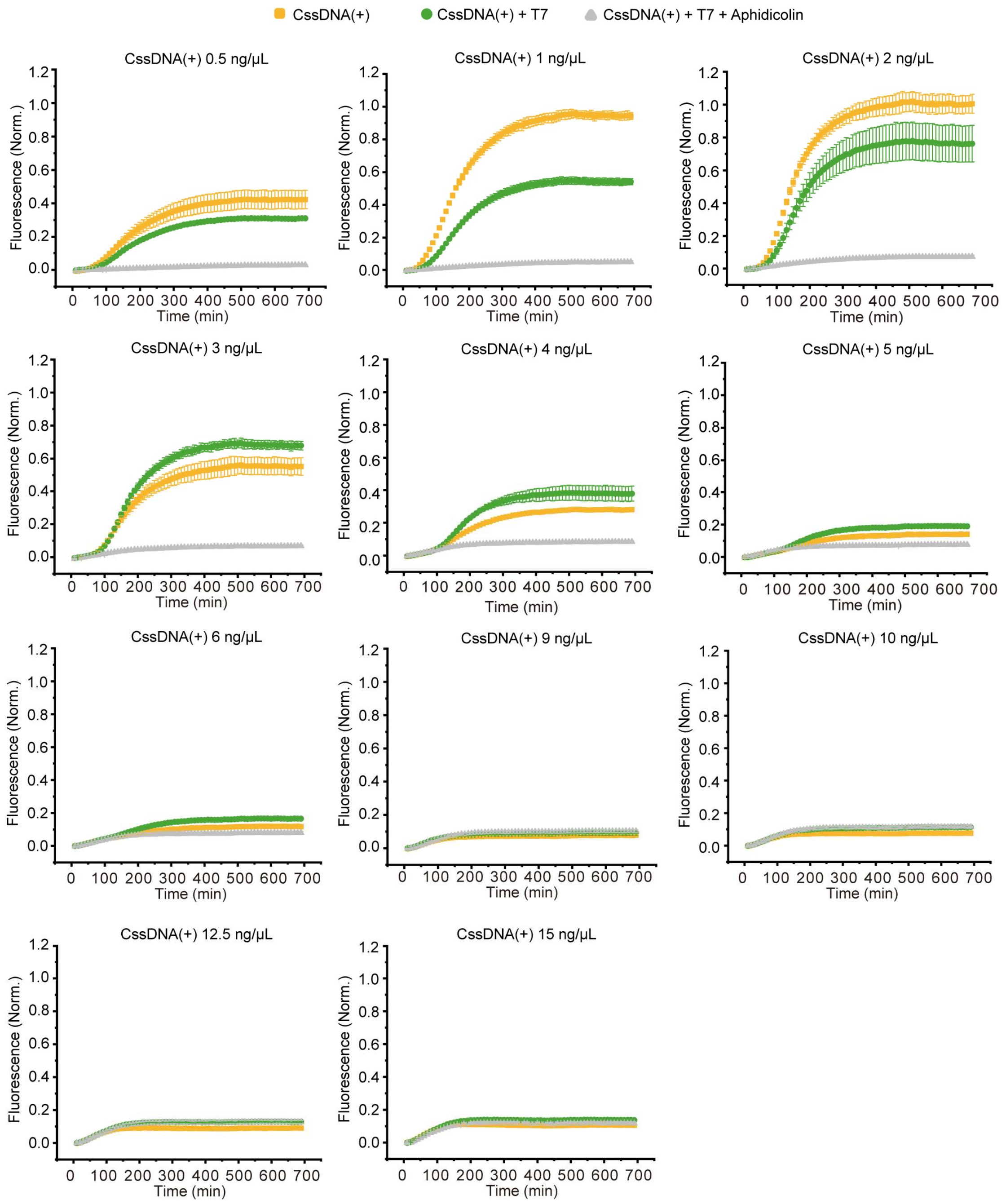

Supplementary Figure 20. Changes of the fluorescence signals of CssDNA(+) at different concentrations over time in the presence of T7 complementary strands and in the coexistence of T7 complementary strands and aphidicolin. All fluorescence signals were normalized based on the average fluorescence of CssDNA(+) expression plateau at a concentration of 2 ng/μL. Data were monitored by a microplate reader and are presented as mean ± standard deviation (s.d.) for $n = 3$ biologically independent experiments. Source data are provided as a Source Data file.

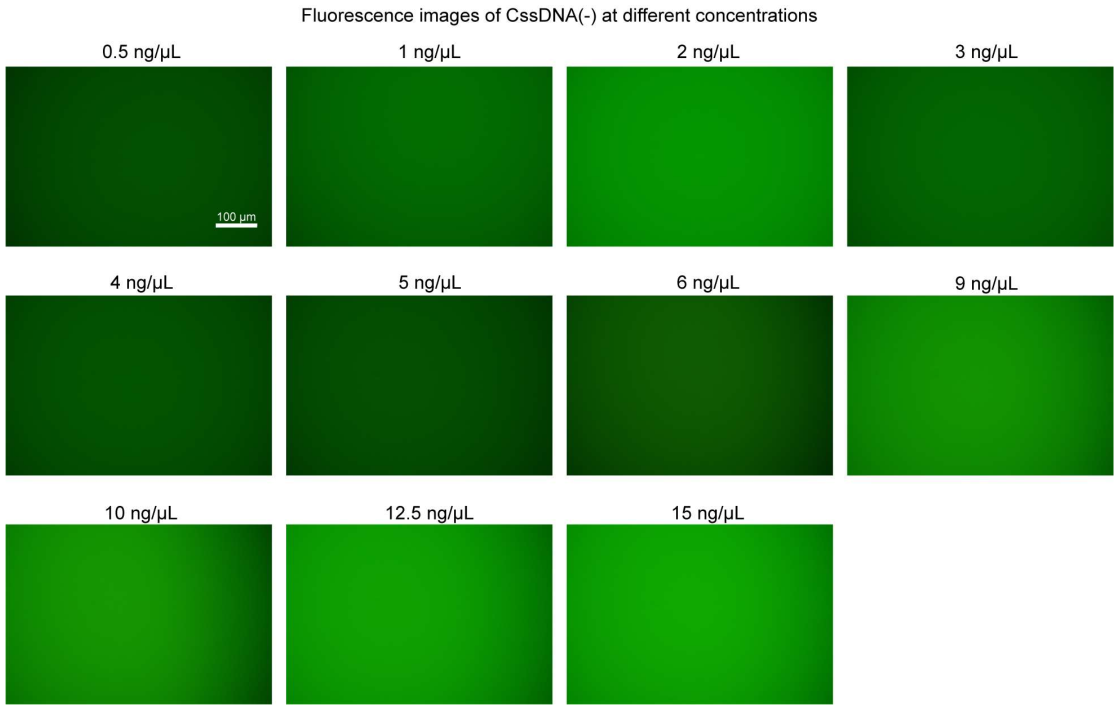

Supplementary Figure 21. Fluorescence images of CssDNA(-) at different concentrations when expression has reached the plateau stage. All images are representative of one of *n* = 3 biologically independent experiments; similar results were observed each time. Scalebar 100 μm.

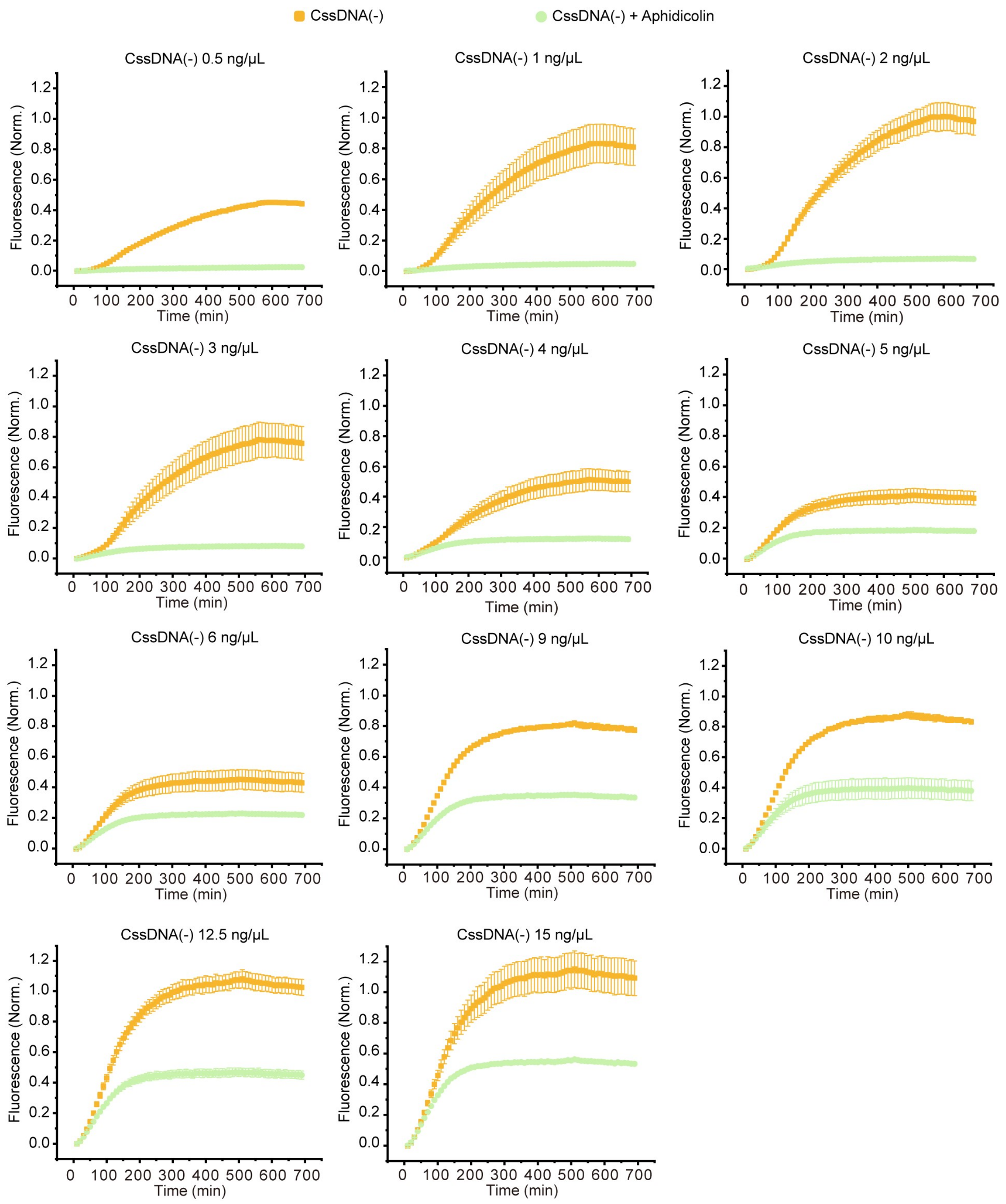

Supplementary Figure 22. Changes of the fluorescence signals of CssDNA(-) at different concentrations over time in the absence and presence of aphidicolin. All fluorescence signals were normalized based on the average fluorescence of CssDNA(-) expression plateau at a concentration of 2 ng/μL. Data were monitored by a microplate reader and are presented as mean ± standard deviation (s.d.) for $n = 3$ biologically independent experiments. Source data are provided as a Source Data file.

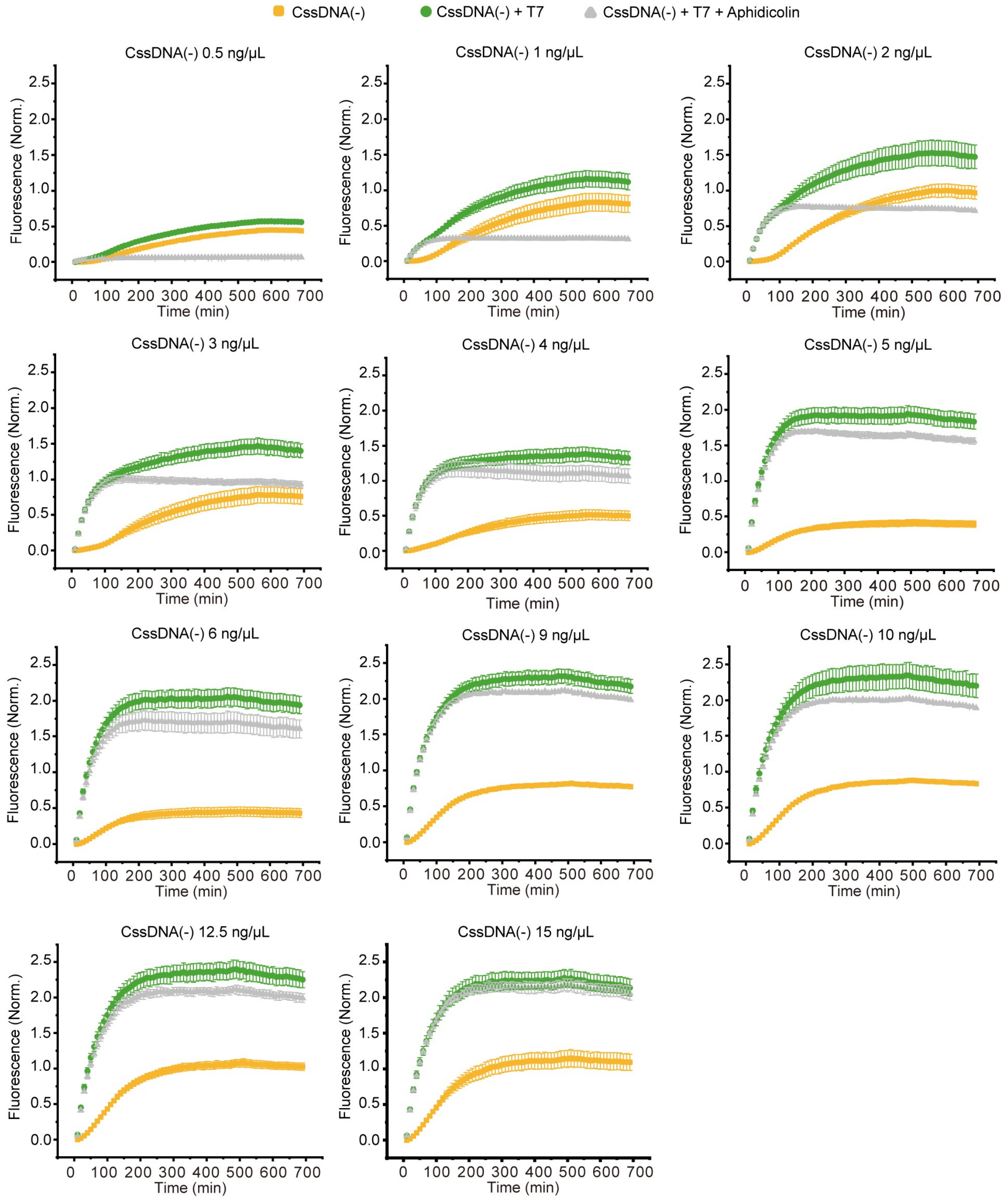

Supplementary Figure 23. Changes of the fluorescence signals of CssDNA(-) at different concentrations over time in the presence of T7 complementary strands and in the coexistence of T7 complementary strands and aphidicolin. All fluorescence signals were normalized based on the average fluorescence of CssDNA(-) expression plateau at a concentration of 2 ng/μL. Data were monitored by a microplate reader and are presented as mean ± standard deviation (s.d.) for n = 3 biologically independent experiments. Source data are provided as a Source Data file.

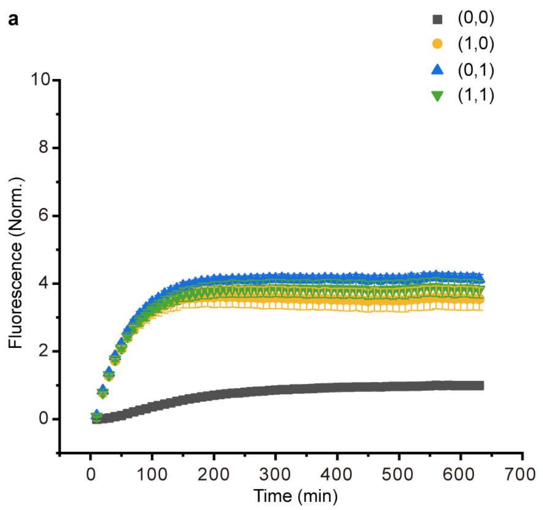

Supplementary Figure 24. Fluorescence signal curves of the OR gate corresponding to different input combinations in the absence (**a**) and presence (**b**) of aphidicolin. All fluorescence signals were normalized according to the fluorescence intensity of the corresponding initial gate structure expression plateau under no input conditions. Data were monitored by a microplate reader and are presented as mean ± standard deviation (s.d.) for $n = 3$ biologically independent experiments. Source data are provided as a Source Data file.

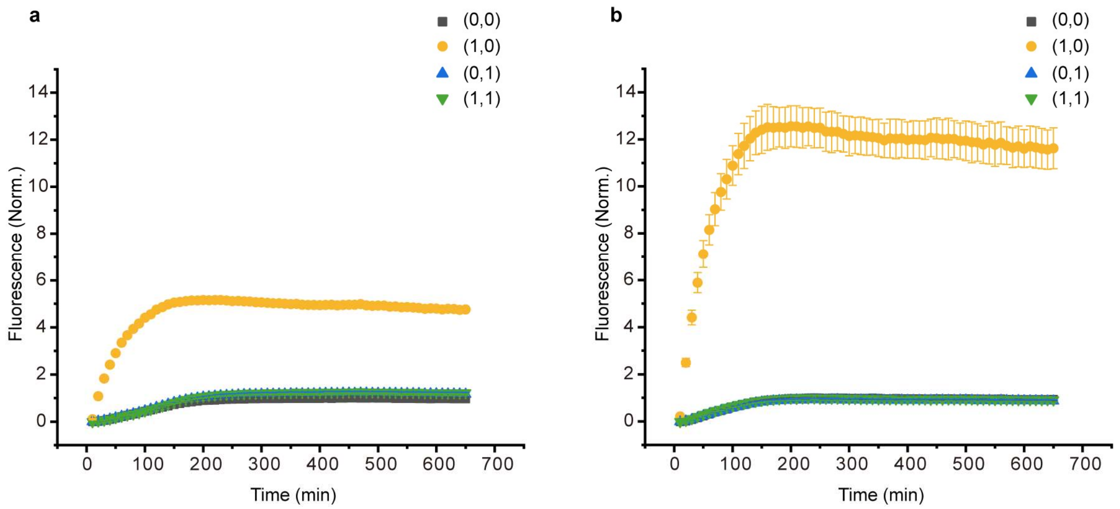

Supplementary Figure 25. Fluorescence signal curves of the INH gate corresponding to different input combinations in the absence (**a**) and presence (**b**) of aphidicolin. All fluorescence signals were normalized according to the fluorescence intensity of the corresponding initial gate structure expression plateau under no input conditions. Data were monitored by a microplate reader and are presented as mean ± standard deviation (s.d.) for $n = 3$ biologically independent experiments. Source data are provided as a Source Data file.

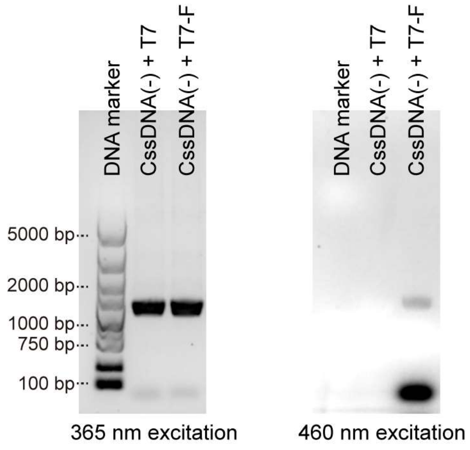

Supplementary Figure 26. 1% agarose gel analysis of CssDNA(-) and its complementary strand assembly. The T7 complementary strands with and without FAM fluorophores were used to bind CssDNA(-) as the complementary strand is too short to be distinguished in agarose gel. The same gel was imaged under two excitation wavelengths, 365 nm and 460 nm. Under 460 nm excitation, a band appeared at the same position as under 365 nm excitation, indicating CssDNA(-) hybridized to T7 complementary strand. The band that migrated rapidly appeared under 460 nm excitation represented the free T7 complementary strand labelled by FAM fluorophores.

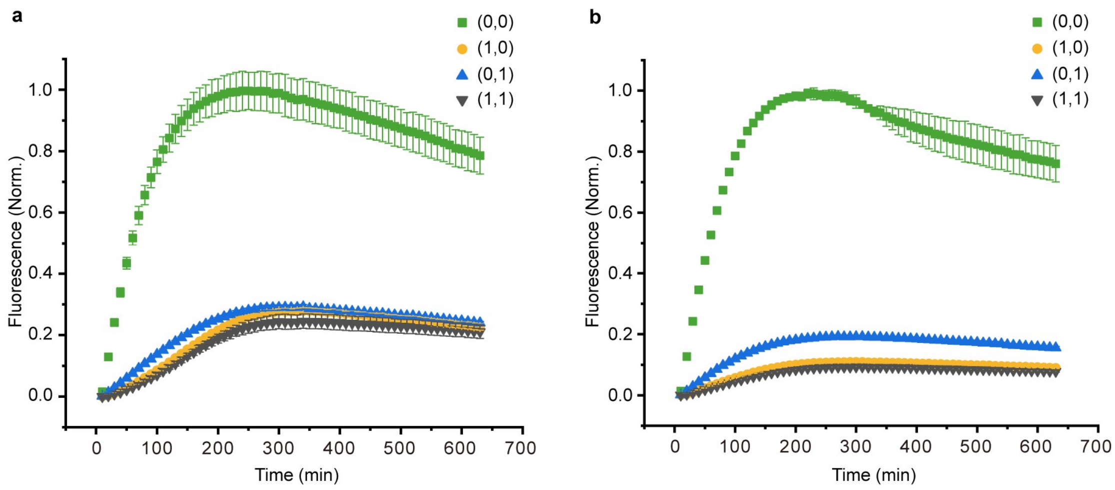

Supplementary Figure 27. Fluorescence signal curves of the NOR gate corresponding to different input combinations in the absence (**a**) and presence (**b**) of aphidicolin. All fluorescence signals were normalized according to the fluorescence intensity of the corresponding initial gate structure expression plateau under no input conditions. Data were monitored by a microplate reader and are presented as mean ± standard deviation (s.d.) for $n = 3$ biologically independent experiments. Source data are provided as a Source Data file.

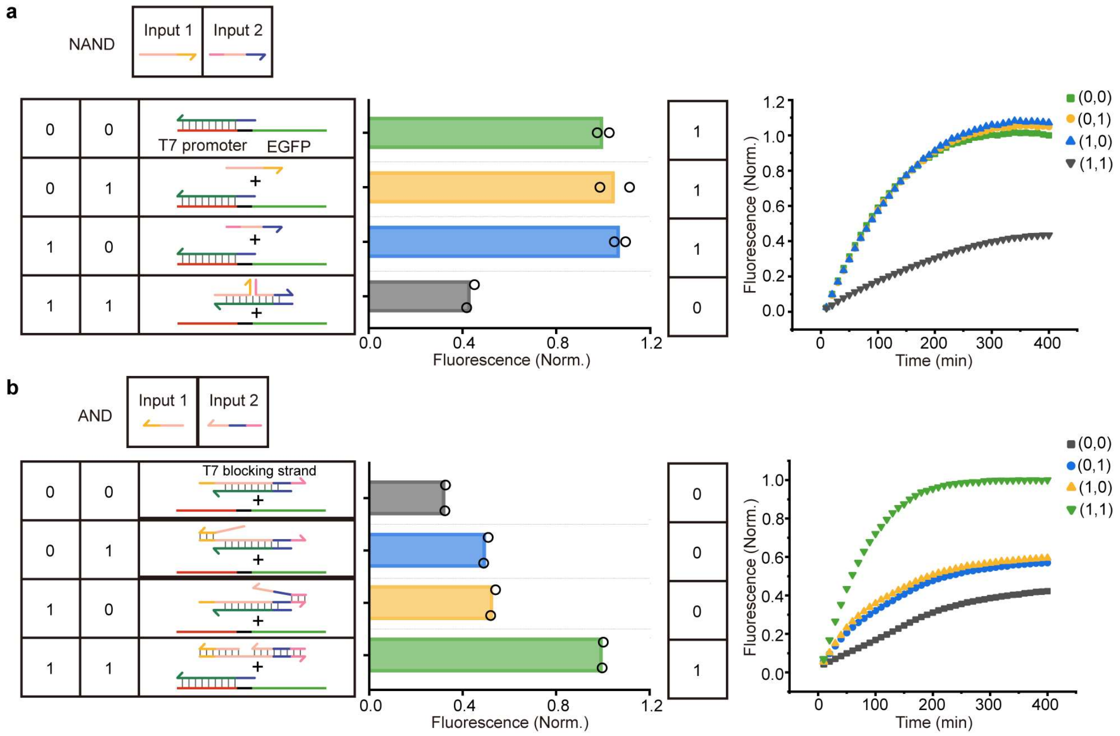

Supplementary Figure 28. Construction of logic gates using CssDNA as a logic element. **a, b.** Schematic and fluorescence signals of two-input logic gates, including NAND (**a**) and AND (**b**) under different input combinations. All fluorescence signals were normalized according to the fluorescence intensity of the corresponding gate structure expression plateau when the output was 1. Data collected in **a** and **b** were monitored by a microplate reader and are presented as means with individual data points overlaid, representing n = 2 biologically independent experiments. Source data are provided as a Source Data file.

To construct the NAND gate, we obtained the initial gate structure by annealing the CssDNA(-) with a longer T7 complementary strand that had toehold sequence at the 5' end. Each input has two parts, the first part is partially complementary to T7 complementary strand, and the second part is partially complementary to the other input. None of them can displace the T7 complementary strands from the initial gate structure. Fluorescence was high either in the absence of an input or in the presence of only one input. When both inputs are present, they can form three-way structure with T7 complementary strand, releasing CssDNA(-) from the initial gate structure (Supplementary Fig. 28a).

For the AND gate, a specially designed double-stranded DNA was introduced into the initial gate. One of the strands is a T7 complementary strand, the other is a T7 blocking strand with the same sequence as CssDNA(-). The input was designed to complement the T7 blocking strand. When there are no inputs or one of the inputs exists, T7 complementary strand was blocked and the fluorescence is low. In the presence of both inputs, T7 complementary strand can be released and the fluorescence is elevated (Supplementary Fig. 28b). The higher background is due to the input sequence being the same as T7 complementary strand.

Supplementary Tables

Supplementary Table 1. Sequences of primers used for plasmids construction.

Primer	Sequence 5' - 3'
Primer-F(+)	TTTGCCGATTTCGGGGTACCGGTGATGTCGGCGATATAGGC
Primer-R(+)	GCTACAGGGCGCGTGGATCCTTATTGCTCAGCGGTGGCA
Primer-F(-)	TTTTGCCGATTTCGGGGTACCTTATTGCTCAGCGGTGGCA
Primer-R(-)	CGCTACAGGGCGCGTGGATCCGGTGATGTCGGCGATATAGGC

Supplementary Table 2. Sequences of CssDNA(+) and CssDNA(-).

CsssDNA(+)
5'- 3'<br>AACAACACTCAACCCTATCTCGGGCTATTCTTTTGATTTATAAGGGATTTTGCCGA<br>TTTCGGGGTACCGGTGATGTCGGCGATATAGGCGCCAGCAACCGCACCTGTGGCGCC<br>GGTGATGCCGGCCACGATGCGTCCGGCGTAGAGGATCGAGATCTCGCGAAATTAATA<br>CGACTCACTATAGGGAAAAAAGAAATCTCTCAAGCTGAAATTAAACCAAAACTCTA<br>ATATAAGAAAAAAAAAATAGAAAGGTATTTTTACAACAATTACCAACAACAACAAACA<br>ACAAACAACATTACAATTACTATTTACAATTACAAAAAAAAAAAATGATCACCGAAA<br>CCTCTTCTCCATTCAGATCTATCTTCTCTCACTCTGGTAAGCACCACCACCACCACCA<br>CCACCACGGTTCTGGTGGATCCAGCCATGGAGGAAGCTTGGTTTCTAAGGGTGAAG<br>AATTGTTCACCGGTGTTGTTCCAATCTTGGTTGAATTGGACGGTGACGTTAACGGTC<br>ACAAGTTCTCTGTTAGAGGTGAAGGTGAAGGTGACGCTACCAACGGTAAGTTGACC<br>TTGAAGTTCATCTGTACCACCGGTAAGTTGCCAGTTCCATGGCCAACCTTGGTTACC<br>ACCTTGACCTACGGTGTTCAATGTTTCTCTAGATACCCAGACCACATGAAGCAACAC<br>GACTTCTTCAAGTCTGCTATGCCAGAAGGTTACGTTCAAGAAAGAACCATCTCTTTC<br>AAGGACGACGGTACCTACAAGACCAGAGCTGAAGTTAAGTTCGAAGGTGACACCTT<br>GGTTAACAGAATCGAATTGAAGGGTATCGACTTCAAGGAAGACGGTAACATCTTGG<br>GTCACAAGTTGGAATACAACTTCAACTCTCACAACGTTTACATCACCGCTGACAAGC<br>AAAAGAACGGTATCAAGGCTAACTTCAAGATCAGACACAACGTTGAAGACGGTTCT<br>GTTCAATTGGCTGACCACTACCAACAAAACACCCCAATCGGTGACGGTCCAGTTTT<br>GTTGCCAGACAACCACTACTTGTCTACCCAATCTAAGTTGTCTAAGGACCCAAACGA<br>AAAGAGAGACCACATGGTTTTGTTGGAATTCGTTACCGCTGCTGGTATCACCTTGGG<br>TATGGACGAATTGTACAAGTAAATAAGGATTAATTACTTGGATGCCAATAAAAAAAA<br>AAAAGCGACATAGCCCGAACTCGACGATCCGGCTGCTAACAAAGCCCGAAAGGAA<br>GCTGAGTTGGCTGCTGCCACCGCTGAGCAATAAGGATCCACGCGCCCTGTAGCGGC<br>GCATTAAGCGCGGCGGGTGTGGTGGTTACGCGCAGCGTGACCGCTACACTTGCCAG<br>CGCCCTAGCGCCCGCTCCTTTCGCTTTCTTCCCTTCCTTTCTCGCCACGTTCGCCGGC<br>TTTCCCCGTCAAGCTCTAAATCGGGGGCTCCCTTTAGGGTTCCGATTTAGTGCTTTAC<br>GGCACCTCGACCCCAAAAAACTTGATTTGGGTGATGGTTCACGTAGTGGGCCATCGC<br>CCTGATAGACGGTTTTTCGCCCTTTGACGTTGGAGTCCACGTTCTTTAATAGTGGACT<br>CTTGTTCCAAACTGGTT
CssDNA(-)

5’- 3’
AACAACACTCAACCCTATCTCGGGCTATTCTTTTGATTTATAAGGGATTTTGCCGA
TTTCGGGGTACCTTATTGCTCAGCGGTGGCAGCAGCCAACTCAGCTTCCTTTCGGGC
TTTGTTAGCAGCCGGATCGTCGAGTTCGGGCTATGTCGCTTTTTTTTTTTTATTGGCAT
CCAAGTAATTAATCCTTATTTACTTGTACAATTCGTCCATACCCAAGGTGATACCAGC
AGCGGTAACGAATTCCAACAAAACCATGTGGTCTCTCTTTTCGTTTGGGTCCTTAGA
CAACTTAGATTGGGTAGACAAGTAGTGGTTGTCTGGCAACAAAACTGGACCGTCAC
CGATTGGGGTGTTTTGTTGGTAGTGGTCAGCCAATTGAACAGAACCGTCTTCAACGT
TGTGTCTGATCTTGAAGTTAGCCTTGATACCGTTCTTTTGCTTGTCAGCGGTGATGTA
AACGTTGTGAGAGTTGAAGTTGTATTCCAACTTGTGACCCAAGATGTTACCGTCTTC
CTTGAAGTCGATACCCTTCAATTCGATTCTGTTAACCAAGGTGTCACCTTCGAACTTA
ACTTCAGCTCTGGTCTTGTAGGTACCGTCGTCCTTGAAAGAGATGGTTCTTTCTTGA
ACGTAACCTTCTGGCATAGCAGACTTGAAGAAGTCGTGTTGCTTCATGTGGTCTGGG
TATCTAGAGAAACATTGAACACCGTAGGTCAAGGTGGTAACCAAGGTTGGCCATGG
AACTGGCAACTTACCGGTGGTACAGATGAACTTCAAGGTCAACTTACCGTTGGTAGC
GTCACCTTCACCTTCACCTCTAACAGAGAACTTGTGACCGTTAACGTCACCGTCCAA
TTCAACCAAGATTGGAACAACACCGGTGAACAATTCTTCACCCTTAGAAACCAAGC
TTCCTCCATGGCTGGATCCACCAGAACCGTGGTGGTGGTGGTGGTGGTGCTTAC
CAGAGTGAGAGAAGATAGATCTGAATGGAGAAGAGGTTTCGGTGATCATTTTTTTT
TTTGTAATTGTAAATAGTAATTGTAATGTTGTTTGTTGTTTGTTGTTGTTGGTAATTGT
TGTAAAAATACCTTTCTATTTTTTTTTCTTATATTAGAGTTTTGGTTTAATTTCAGCTTG
AGAGATTTCTTTTTTCCCTATAGTGAGTCGTATTAATTTCGCGAGATCTCGATCCTCTA
CGCCGGACGCATCGTGGCCGGCATCACCGGCGCCACAGGTGCGGTTGCTGGCGCCT
ATATCGCCGACATCACCGGATCCACGCGCCCTGTAGCGGCGCATTAAGCGCGGCGGG
TGTGGTGGTTACGCGCAGCGTGACCGCTACACTTGCCAGCGCCCTAGCGCCCGCTC
CTTTCGCTTTCTTCCCTTCCTTTCTCGCCACGTTCGCCGGCTTTCCCCGTCAAGCTCT
AAATCGGGGGCTCCCTTTAGGGTTCCGATTTAGTGCTTTACGGCACCTCGACCCCAA
AAAACTTGATTTGGGTGATGGTTCACGTAGTGGGCCATCGCCCTGATAGACGGTTTT
TCGCCCTTTGACGTTGGAGTCCACGTTCTTTAATAGTGGACTCTTGTTCCAAACTGG
TT

Supplementary Table 3. Sequences of complementary strands of CssDNA.

Name	Sequence 5’- 3’
CssDNA(+)-T7	CCTATAGTGAGTCGTATTA
CssDNA(-)-T7	TAATACGACTCACTATAGG
T7-27nt	CGCGAAATTAATACGACTCACTATAGG
T7-23nt	AAATTAATACGACTCACTATAGG
T7-19nt	TAATACGACTCACTATAGG
T7-17nt	TAATACGACTCACTATA
T7-13nt	ATACGACTCACTA
T7-9nt	ACGACTCAC
P1	GCCAGCAACCGCACCTGTG
P2	GCGCCGGTGATGCCGGCCA
P3	CGATGCGTCCGGCGTAGAG

P4	GATCGAGATCTCGCGAAAT
P5	TAATACGACTCACTATAGG
P6	GAAAAAAGAAATCTCTCAA
P7	GCTGAAATTAAACCAAAAC
P8	TCTAATATAAGAAAAAAAA
P9	ATAGAAAGGTATTTTTACA
P10	ACAATTACCAACAACAACA
P11	AACAACAAACAACATTACA
P12	ATTACTATTTACAATTACA
P13	AAAAAAAAAAATGATCACC
P14	GAAACCTCTTCTCCATTCA

Supplementary Table 4. Sequences of DNA strands for the construction of logic gates.

Name	Sequence 5’- 3’
OR (input 1)	AAATTAATACGACTCACTATAGG
OR (input 2)	TAATACGACTCACTATAGG
INH (input 1)	ATGCCTGCTGTAATACGACTCACTATAGG
INH (input 2)	CCTATAGTGAGTCGTATTACAGCAGGCAT
NOR (input 1)	CCTATAGTGAGTCGTATTA CAGCAGGCAT
NOR (input 2)	CGATACGAGTCCTATAGTGAGTCGTATTACAG
NOR (longer T7)	ATGCCTGCTGTAATACGACTCACTATAGGACTCGTATCG
NAND (input 1)	CCATTACTCTACCTTATTACAGCAGGCAT
NAND (input 2)	CCTATAGTGAGTCGAGGTAGAGTAATGG
NAND (longer T7)	ATGCCTGCTGTAATACGACTCACTATAGG
AND (input 1)	ACTATAGATGCCTGCTG
AND (input 2)	CTGCATTGATGCCTGCTGTAATACG
AND (T7 complementary)	ATGCCTGCTGTAATACGACTCACTATAG
AND (T7 blocking)	CAGCAGGCATCTATAGTGAGTCGTATTACAGCAGGCATCAATGCAG

Supplementary References

1. Tang, L., Tian, Z., Cheng, J. et al. Circular single-stranded DNA as switchable vector for gene expression in mammalian cells. *Nat. Commun.* **14**, 6665 (2023).
